# A Pilot Study Comparing the Effects of Concurrent and Terminal Visual Feedback on Standing Balance in Older Adults

**DOI:** 10.3390/s22082826

**Published:** 2022-04-07

**Authors:** Jamie Ferris, Vincent J. Barone, Noel C. Perkins, Kathleen H. Sienko

**Affiliations:** Department of Mechanical Engineering, University of Michigan, Ann Arbor, MI 48109, USA; jcferris@umich.edu (J.F.); vbarone@umich.edu (V.J.B.); ncp@umich.edu (N.C.P.)

**Keywords:** balance, postural control, feedback, visual feedback, concurrent, terminal, older adult

## Abstract

While balance training with concurrent feedback has been shown to improve real-time balance in older adults, terminal feedback may simplify implementation outside of clinical settings. Similarly, visual feedback is particularly well-suited for use outside the clinic as it is relatively easily understood and accessible via ubiquitous mobile devices (e.g., smartphones) with little additional peripheral equipment. However, differences in the effects of concurrent and terminal visual feedback are not yet well understood. We therefore performed a pilot study that directly compared the immediate effects of concurrent and terminal visual feedback as a first and necessary step in the future design of visual feedback technologies for balance training outside of clinical settings. Nineteen healthy older adults participated in a single balance training session during which they performed 38 trials of a single balance exercise including trials with concurrent, terminal or no visual feedback. Analysis of trunk angular position and velocity features recorded via an inertial measurement unit indicated that sway angles decreased with training regardless of feedback type, but sway velocity increased with concurrent feedback and decreased with terminal feedback. After removing feedback, training with either feedback type yielded decreased mean velocity, but only terminal feedback yielded decreased sway angles. Consequently, this study suggests that, for older adults, terminal visual feedback may be a viable alternative to concurrent visual feedback for short duration single-task balance training. Terminal feedback provided using ubiquitous devices should be further explored for balance training outside of clinical settings.

## 1. Introduction

Balance performance deteriorates with age, leading to increased fall risk and fall prevalence in healthy older adults [1]. Poor balance confidence and fear of falling are also associated with increased anxiety [2,3,4], depression [2,3], and institutionalization [4,5], and with decreased activity [3,6,7], social participation [7,8], and quality of life [3,7].

Balance training, which is traditionally performed in a clinical setting with the instruction of a physical therapist, has been shown to improve balance performance for older adults as measured by clinical tests of balance (e.g., Berg Balance Scale) and sway metrics (e.g., center of mass displacements) [9,10,11]. However, the cost and availability of balance therapy [12,13], for either therapeutic or preventative use, limits access to balance training. For example, it is predicted that by 2030, the United States alone will have a shortage of 140,000 physical therapists, resulting in the majority of patients having difficulty accessing necessary physical therapy services [12]. 

Balance training technologies designed to be used outside of clinical settings may therefore increase access to preventative and therapeutic balance training. However, balance training without a physical therapist’s supervision has been shown to be less effective than supervised training [14,15]. This shortcoming may be mitigated by providing feedback during balance training. Feedback systems typically comprise a combination of sensors and displays [16]. Previous studies have explored the use of accelerometers or inertial measurement units (IMUs) [10,17,18,19,20,21,22,23], plantar pressure [10,18,24] or force sensors [17,18,21,24,25,26,27,28], cameras [17,26,29,30,31], or electromyography (EMG) [24] to measure kinematics, kinetics, or muscle activity information about postural sway and gait dynamics, while feedback has been provided via visual, tactile, auditory, and multimodal displays [10,16,24].

Feedback devices have been used as real-time balance aids and as tools to augment balance training programs [32]. Over the past 30 years, numerous studies have shown that feedback is effective at improving real-time quiet and perturbed standing balance, i.e., balance while the feedback is being provided [20,21,33,34,35,36,37,38,39]. More recently, a limited number of studies have compared the effects of balance training programs with and without feedback, with a subset of such studies showing additional benefits of training with feedback. In a 2018 review, Gordt et al. (2017) examined eight studies assessing the effects of wearable-sensor-based feedback during single- or multi-day balance and gait training for individuals with various balance disorders and found evidence of greater reductions in postural sway after training with feedback than after conventional balance training [10]. Alhasan et al. (2017) reviewed five studies conducting multiple sessions of standing balance training with visual feedback among older adults and concluded that training with visual feedback is likely to lead to greater improvements in clinical outcome measures and/or trunk sway compared to balance training without feedback or no intervention [25]. Similarly, Mak et al. (2021) examined 17 studies on the same topic and found there to be benefits of visual feedback on clinical balance measures and/or trunk sway, with seven out of eight studies finding training with visual feedback to yield greater improvements than no intervention, and three out of three studies finding training with visual feedback to yield greater improvements than placebo interventions [17]. In an examination of vibrotactile feedback, Bao et al. (2018) reported improved clinical outcome measures in older adults following an eight-week home-based balance training program using a smartphone-based vibrotactile feedback system, and the improvements were significantly greater for a subset of the clinical metrics assessed compared to improvements for a control group completing the same training without feedback [19]. Similarly, uncontrolled studies by Basta et al. (2011) and Rossi-Izquierdo et al. (2013) reported reduced trunk sway and improved clinical outcome measures after two weeks of balance training with vibrotactile feedback in people with various balance disorders or Parkinson’s disease, respectively, and a controlled study by Brugnera et al. (2015) reported improved clinical outcome measures after two weeks of training with vibrotactile feedback and no improvement after two weeks of training without feedback in people with Parkinson’s disease [40,41,42]. Finally, studies examining balance exergaming with multimodal (auditory and visual) feedback enabled by the Xbox^®^ Kinect have shown that balance training with feedback is more effective than conventional training at improving clinical outcome measures and measures of postural sway [30,31]. Although there are examples in the literature that report additional improvement when balance training is accompanied by feedback compared to training alone, such as a 2010 review that found that nine out of 13 controlled studies involving older adults reported greater improvements in clinical outcome measures and measures of postural sway with feedback-augmented balance training than training without feedback (and none of the studies reported greater improvements without feedback), there have not been any large-scale randomized controlled trials reported to date and the impact of the improvements on fall rates have not been assessed [24].

While numerous feedback modalities have yielded benefits during balance training, some training systems require specialized equipment (e.g., vibrating actuators), which may limit access for certain prospective users. Visual feedback offers the benefits of being relatively easily understood [24], able to encode both spatial and temporal information (e.g., sway position and velocity) [43], and immediately available via ubiquitous mobile devices (e.g., smartphones, tablets) with little additional peripheral equipment. Terminal visual feedback specifically (i.e., feedback provided after the balance exercise is completed) can be achieved using a single mobile device and therefore offers a simpler and less costly alternative that also extends to additional balance exercises (e.g., when eyes are closed).

However, the effects of terminal visual feedback on balance training performance are not well understood. Extended balance training with concurrent visual feedback (i.e., feedback provided in real-time during the balance exercise) has been shown to result in greater balance improvements than training without feedback [17,25]. Similarly, a single session of balance training with concurrent visual feedback has been shown to yield greater sway reductions than training without feedback under certain conditions, including reducing the root mean square (RMS) of the center of pressure for older adults on a compliant surface with feet displayed at a 30° angle [21]. However, it also requires that a properly placed screen be visible for the duration of each exercise. Because the timing of feedback can impact its efficacy [44,45,46], the effects of concurrent feedback during balance training may not translate to terminal feedback. For simple tasks, concurrent feedback may result in immediate balance improvement, but the user may also become reliant on the feedback, leading to poor retention of benefits. Terminal feedback, on the other hand, may result in smaller immediate balance improvements but superior retention [16,47]. However, because the ‘simplicity’ of a task is difficult to assess as it relies upon both the task complexity and user skill level, it is difficult to predict the effect of terminal feedback on balance training in older adults [47].

Goodwin and Goggin (2018) compared the use of terminal visual feedback and a *combination* of concurrent and terminal visual feedback on older adults’ dynamic balance and found that both types of feedback improved balance performance during a single training session [48]. Greater retention of balance performance was achieved following training with terminal feedback compared to a combination of concurrent and terminal feedback. However, to our knowledge no studies have directly examined terminal visual feedback or compared solely concurrent to solely terminal visual feedback. 

We therefore sought to extend the existing literature by performing a pilot study that directly compared the effects of training with concurrent or terminal visual feedback on older adults completing a common balance exercise. We evaluated the effects by quantifying the RMS and velocity of the postural sway angle. This preliminary study represents a necessary first step in a series of studies that would be needed to develop a balance training technology that leverages a single mobile device to provide terminal visual feedback outside of clinical settings.

## 2. Materials and Methods

### 2.1. Participants

Nineteen older adult participants were recruited through the University of Michigan Health Research website (demographics shown in Table 1). All participants self-reported that they were in good general health (i.e., medically stable, no frequent back or lower extremity pain, no severe visual impairment, no history of fainting) and had no muscular or neurological disorders that would affect balance performance. Healthy older adults were included in this pilot study because they were readily accessible, could complete a large number of trials within a single session, and would potentially use a home-based balance training technology for preventative balance training. The study was approved by the University of Michigan Institutional Review Board (study HUM00015990), and all participants gave written informed consent in accordance with the Helsinki Declaration.

### 2.2. Protocol

All participants partook in a single-day balance training session in which they stood on a foam pad (regular balance pad, 50 × 41 × 6 cm^3^, Airex AG, Sins, Switzerland) with their feet together. This balance exercise was selected because it was sufficiently challenging such that older adults could benefit from feedback, but was not so difficult that they would routinely step out of position during a 30-s trial [21]. The participants were barefoot and wore comfortable clothing, and they were instructed to stand quietly while maintaining the pose. They were told to place and move their arms however they liked, but to not touch the support in front of them unless they needed to do so to prevent a fall. No additional instructions were given regarding balance strategy.

For the duration of training, participants wore a single (six degree of freedom) IMU (MTx, XSens Inc., Eschende, The Netherlands) on an elastic belt approximately positioned over the L3 vertebrae level dorsal to the spine. IMU data (acceleration and angular rate) were collected at 100 Hz using custom software and the trunk sway angles were extracted using XSens’ proprietary sensor fusion algorithm. From among the options for measuring balance performance (e.g., center of pressure displacements, clinical measures such as the Berg Balance Score), IMUs were selected because they can be found in smartphones and are therefore well-suited for accessible, at-home training systems. 

**Figure 1:**
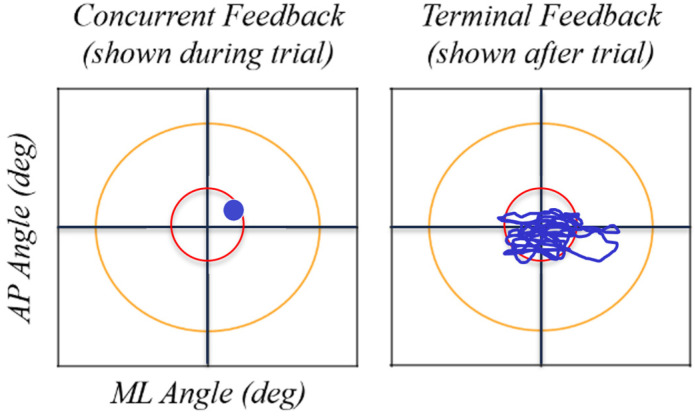
Example feedback displays for concurrent and terminal feedback.

Using custom software and live-streamed or recorded trunk sway data, visual feedback was displayed on a projector screen placed 10 feet in front of the participant with the center of the display approximately level with the participant’s eyes. A pair of horizontal and vertical axes shown at all times represented the medial-lateral (ML) and anterior-posterior (AP) sway angles, respectively. Concurrent feedback was displayed as a single cursor on the screen denoting the current ML and AP angles of the participant, and terminal feedback was displayed before the trial as a stabilogram illustrating all ML and AP tilt angle pairs from the trial immediately preceding the current trial (see example feedback displays in Figure 1). Participants were shown examples of each type of feedback and told what the target represented. Then, prior to beginning training with each type of feedback, participants practiced with that feedback type while standing with a shoulder width stance on a firm surface (a stance and surface different than the test conditions) for no more than one minute. Adopting a different exercise than the training exercise allowed the participants to acclimate to the feedback without beginning to learn the training balance exercise prior to the start of data collection. No additional instructions were given regarding how to interpret or apply the feedback.

**Figure 2:**
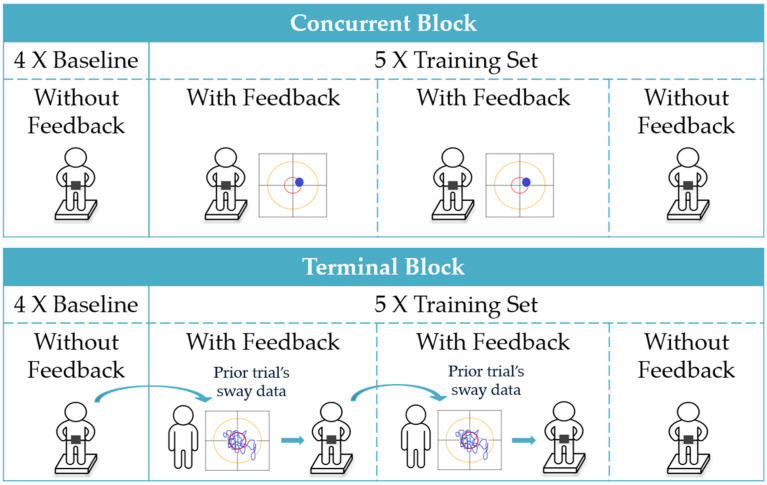
Trial structure for concurrent and terminal feedback blocks. Each block (terminal and concurrent) consisted of four baseline trials with no feedback followed by five sets of three 30-s training trials, with feedback provided during the first and second trials and no feedback provided during the third trial.

Feedback was provided using a crossover design wherein participants completed a block of training with concurrent visual feedback and a block of training with terminal visual feedback, where order was randomly assigned by alternating between subsequent participants. Participants completed a total of 38 trials during the training session, performing 19 trials within the concurrent feedback block and 19 trials within the terminal feedback block (see Figure 2). So as to avoid the effects of fatigue, the total number of training trials was selected to yield a training duration less than the typical duration of a single-day balance therapy or at-home balance training session; the active training duration was 19 min compared to an optimal training duration of 31–45 min [9]. 

Each block consisted of four baseline trials with no feedback followed by five sets of three 30-s training trials, with feedback provided during the first and second trials and no feedback provided during the third trial (i.e., feedback provided 2/3 or 67% of the time). While the optimal ratio of trials with feedback to trials without feedback is unknown, providing feedback on fewer than 100% of trials (i.e., including some trials without feedback) has been shown to encourage integration and motor learning [16,49,50,51,52,53]. Participants took a short break of approximately 5 min between blocks. Participants 1 through 7 did not complete the last (19th) trial in the terminal block and the last baseline (4th) trial in the concurrent block due to a change in experimental design to better balance the baseline trials. As a result, seven participants completed 36 trials and 12 participants completed 38 trials, and three of the total 708 trials were excluded from the analysis due to data loss. 

### 2.3. Data Analysis

The IMU data recorded using the custom software were also imported to MATLAB™ (Mathworks, Natick, MA, USA) where the angles were filtered using a second order Butterworth filter with a 2 Hz cutoff frequency and then analyzed. Performance features calculated from the IMU data included the RMS of the sway angle from vertical (Phi RMS, degrees; Phi_Angle^2^ = AP_Angle^2^ + ML_Angle^2^), RMS in the AP direction (AP RMS, degrees), RMS in the ML direction (ML RMS, degrees), mean sway velocity (MV, degrees/s), path length as computed by the sum of the magnitude of the differences between sway data points (PL, degrees), and area of a 95th percentile confidence interval elliptical fit to the sway data (i.e., elliptical area; EA, degrees^2^) [11,27]. These features were calculated as

(1) $$\mathrm{Phi}\;\mathrm{RMS}=\sqrt{\frac{1}{J}\sum_{j=1}^{J}{\left(\mathrm{AP}\left[j\right]-\frac{1}{J}\sum^{\;}\mathrm{AP}\left[j\right]\right)}^{2}+{\left(\mathrm{ML}\left[j\right]-\frac{1}{J}\sum^{\;}\mathrm{ML}\left[j\right]\right)}^{2}}$$

(2) $$\mathrm{AP}\;\mathrm{RMS}=\sqrt{\frac{1}{J}\sum_{j=1}^{J}{\left(\mathrm{AP}\left[j\right]-\frac{1}{J}\sum^{\;}\mathrm{AP}\left[j\right]\right)}^{2}}$$

(3) $$\mathrm{ML}\;\mathrm{RMS}=\sqrt{\frac{1}{J}\sum_{j=1}^{J}{\left(\mathrm{ML}\left[j\right]-\frac{1}{J}\sum^{\;}\mathrm{ML}\left[j\right]\right)}^{2}}$$

(4) $$\mathrm{MV}=\frac{\sum_{j=1}^{J-1}\sqrt{{\left(\mathrm{AP}\left[j+1\right]-\mathrm{AP}\left[j\right]\right)}^{2}+{\left(\mathrm{ML}\left[j+1\right]-\mathrm{ML}\left[j\right]\right)}^{2}}}{T}$$

(5) $$\mathrm{PL}=\sum_{j=1}^{N-1}\sqrt{{\left(\mathrm{AP}\left[j+1\right]-\mathrm{AP}\left[j\right]\right)}^{2}+{\left(\mathrm{ML}\left[j+1\right]-\mathrm{ML}\left[j\right]\right)}^{2}}$$

(6) $$\mathrm{EA}={\pi \;z}_{0.95}^{2}\lambda_{1}^{0.5}\left(\sigma_{\mathrm{AP}\;\mathrm{ML}}^{2}\right)\lambda_{2}^{0.5}\left(\sigma_{\mathrm{AP}\;\mathrm{ML}}^{2}\right)$$

where J refers to the number of data points, AP[j] refers to the jth AP data point, T is the total time elapsed (i.e., the time at which data point J was collected minus the time at which the first data point was collected), z_0.95_ is the z-score for a 95% confidence level and $\lambda_{1}\left(\sigma_{\mathrm{AP}\;\mathrm{ML}}^{2}\right)$ and $\lambda_{2}\left(\sigma_{\mathrm{AP}\;\mathrm{ML}}^{2}\right)$ are the 1st and 2nd eigenvalues of the covariance of the AP and ML data. Based on results from previously published studies involving different modalities of feedback, smaller RMS and EA values typically indicate better balance performance [22,23,28,54], while in some studies MV has increased while participants use concurrent feedback following limited training [23,54].

A single linear mixed-effects regression (LME) was used to compare the changes in performance as a function of training and feedback status. The dependent variable was the log of one performance feature, and all tests were performed with α = 0.05. The maximal model was

ln Feature ~ 1 + Order + Block + TrialNumber ^a^ + ConcurrentFeedbackRemoved + TerminalFeedbackRemoved + Block:TrialNumber ^a^ + Block:Order + ConcurrentFeedbackRemoved:Order + TerminalFeedbackRemoved:Order + Age + Sex + (1|Participant). (7)

The fixed effects include the intercept (‘1’), the order in which the feedback modes were used (Order), a Boolean for being in the concurrent feedback block (‘Block’), the exponentiated number of trials completed after the first baseline (‘TrialNumber ^a^’, considered 0 < *a* ≤ 5 in increments of 0.1), a Boolean for concurrent/terminal feedback being removed (‘ConcurrentFeedbackRemoved’/’TerminalFeedbackRemoved’), age and sex of the participant, and the interaction terms listed. The random intercept is for participant ID (‘1|Participant’), and ‘:’ is used to denote an interaction. This maximal model was then reduced for each feature independently by removing terms that yielded lower Akaike information criteria (AIC) scores and that were not of direct interest (i.e., all terms except ‘TrialNumber’, ‘ConcurrentFeedbackRemoved’, and ‘TerminalFeedbackRemoved’). The included terms are shown in Table 2.

## 3. Results

**Figure 3:**
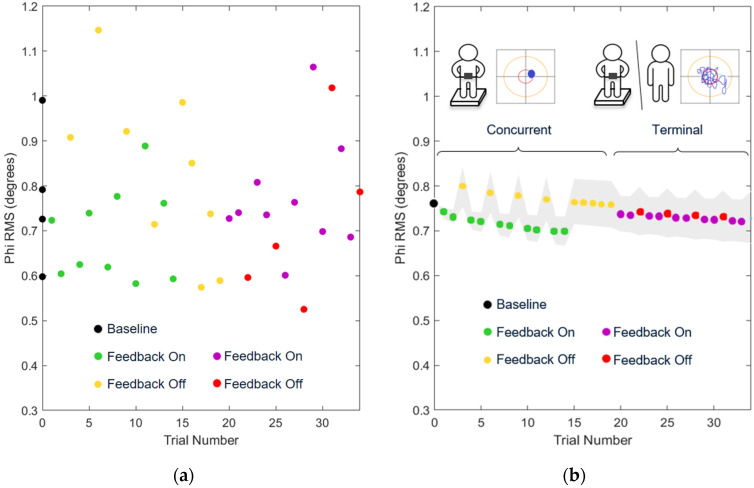
(**a**) Measured Phi RMS for an exemplar participant in Group 1; (**b**) Predicted Phi RMS values and 95% confidence intervals for the same exemplar participant calculated using an LME, where the 95% confidence band included only the effects of direct interest (i.e., ‘TrialNumber ^a^’, ‘ConcurrentFeedbackRemoved’, and ‘TerminalFeedbackRemoved’). Phi RMS decreased significantly and at the same log rate with both types of feedback. Phi RMS increased significantly with the removal of concurrent feedback, but not significantly with the removal of terminal feedback.

Figure 3 shows the measured data as well as the combined effect of all included model terms for the Phi RMS of a representative exemplar participant.

Table 3 reports the significance and effect sizes of individual LME factors. As the number of trials with concurrent feedback increased, sway angle (Phi RMS, AP RMS, ML RMS, EA) decreased while mean velocity (MV) increased (see Table 3). Terminal feedback resulted in the same reduction in sway angle (Phi RMS, AP RMS, EA) as concurrent feedback, but with a decrease in MV.

The effects of removing feedback were significantly smaller after training with terminal compared to concurrent feedback (see Table 3). After concurrent feedback was removed, all features exhibited a significant change in the opposite direction as the effects of training with feedback. In contrast, the differences observed following training with terminal feedback were largely maintained once terminal feedback was removed. 

When comparing the predicted feature values at baseline and the end of the first block, terminal feedback resulted in significant decreases in Phi RMS, AP RMS, and EA while concurrent feedback resulted in no significant changes in the same features (see Table 4). Ultimately, repeated practice with either type of feedback resulted in significant decreases in MV (with terminal yielding a greater decrease; *p* = 0.037, estimate −0.081, [−0.1593, −0.003]) and PL, while only terminal feedback resulted in significant decreases in RMS and EA.

## 4. Discussion

### 4.1. Effects of Balance Training with Feedback While Feedback Was Used

While receiving either type of feedback during a single session of balance training, older adult participants in this study exhibited improved postural steadiness as characterized by RMS and EA. However, participants exhibited decreased mean sway velocity only with terminal feedback.

An increased number of trials with either type of feedback resulted in decreased RMS and EA (Phi RMS *p* < 0.001, AP RMS *p* < 0.001, EA *p* < 0.001), indicating that participants could use concurrent visual feedback and terminal visual feedback to maintain a more stable posture. Although not the focus of this work, these findings are consistent with prior research that has shown decreases in RMS and EA when concurrent feedback was provided as a real-time balance aid [20,21,36,55]. In addition, sway velocity increased with concurrent feedback (*p* = 0.012) and decreased with terminal feedback (*p* < 0.001). Because sway velocity has been linked to increased postural control activity (i.e., amount of balancing activity needed to maintain a given level of postural stability [27,33,56]), we posit that increased postural control activity with concurrent feedback and decreased activity with terminal feedback accompanied the decrease in sway angles [55]. The measured change in sway velocity suggests that participants used concurrent feedback to make many rapid postural corrections while they used terminal feedback to make smaller, slower corrections. Therefore, while training with both types of feedback resulted in decreased sway, concurrent feedback may have elicited postural corrections each time the feedback showed a deviation from center (resulting in many rapid corrections) while terminal feedback may have elicited improvements to a participant’s internal model relating intrinsic feedback to deviations from center [16,47]. These findings align with the guidance hypothesis which predicts that, for simple tasks, concurrent feedback accelerates training during the acquisition phase as knowledge of the outcome can be used from moment to moment to correct errors. In contrast, terminal feedback is predicted to produce less immediate improvement because the delayed feedback can only guide the next balance attempt [16,47]. These findings may also reflect that the terminal feedback conveyed limited velocity information (i.e., characteristics of change in direction, but not speed) as compared to the concurrent feedback. While future studies may investigate the effects of providing additional velocity information via the terminal feedback display, we opted to keep the feedback presentation format as consistent as possible in this pilot study as it was the first in a series of studies needed on the topic.

Comparing these results to prior studies, the concurrent feedback findings align with those reported by Dos Anjos et al. (2016) [55] (i.e., decreased COP EA and MV among adults when receiving concurrent COP feedback while standing with feet together on a firm surface), Halická et al. (2011) (i.e., decreased COP RMS among older adults when receiving concurrent COP feedback while standing with heels together and feet displayed at an angle of 30° on a foam surface) [21], and Dault et al. (2003) (i.e., increased COP MV among older adults when receiving concurrent COP feedback while standing with feet apart on a firm surface) [57]. In contrast, Halická et al. (2011) reported no change in the RMS of the center of mass as measured using accelerometers on the lower back [21], and Dault et al. (2003) reported no reduction in COP sway amplitude [57]. However, these studies included fewer older adult participants and/or fewer trials than the current study, making it potentially difficult to detect the current study’s small effect size.

Because our aim was to compare the effects of balance training with concurrent versus terminal feedback, we conducted a direct comparison of these two feedback types rather than comparing to a control group that trained without feedback. However, feedback has been shown to yield significantly less postural sway (30–50%) when used as a real-time aid compared to balancing without feedback [21,28,33,34,35,36,37,38,39]. Similarly, after an extended training program, training with concurrent visual feedback has yielded greater improvements in clinical balance test scores than training without feedback [17,25]. Therefore, the changes observed here within a single block of balance training with concurrent feedback were likely greater than the changes that would have occurred during training without feedback. Because terminal feedback yielded changes equal to or larger than balance training with concurrent feedback, terminal feedback likely also yielded greater changes than would have occurred without feedback.

### 4.2. Effects of Balance Training with Feedback after Feedback Was Removed

Once either type of feedback was removed after a single session of balance training, older adult participants in this study reduced their mean sway velocity. However, only after a session with terminal feedback did participants exhibit improved postural steadiness as characterized by RMS and EA.

After training with concurrent feedback, RMS and EA values were not significantly different than baseline values (Phi RMS *p* = 0.910, AP RMS *p* = 0.557, ML RMS *p* = 0.732, EA *p* = 0.815), suggesting that participants did not experience a significant amount of motor learning during the single session, a finding that agrees with Wiesmeier et al. (2017) [58]. In contrast, after training with terminal feedback, RMS and EA values were both lower than baseline values (Phi RMS *p* = 0.022, AP RMS *p* = 0.005, EA *p* = 0.013), indicating that some motor learning occurred. These findings align with the guidance hypothesis’ claim that concurrent feedback is detrimental to skill retention because participants grow dependent on artificial feedback and disregard natural feedback while terminal feedback is beneficial for balance skill retention as participants improve their internal balance mechanisms [16,47]. After training, the resulting decrease in MV values relative to baseline values was significantly greater with terminal feedback than concurrent feedback (*p* = 0.037), suggesting that participants achieved better postural control performance with less postural control activity after receiving terminal compared to concurrent feedback [27,33,56]. 

The findings from this study were consistent with some prior studies comparing concurrent and terminal visual feedback on simple or complex tasks, albeit with different tasks and training schedules than employed herein [58]. Ranganathan and Newell (2009) tested a single session of concurrent or terminal visual feedback in a force-production task and found that concurrent feedback facilitated the adoption of different strategies to achieve the goal, but that the improvement was not retained. Unlike the current findings, Ranganathan and Newell found that terminal feedback did not yield improvements; however, they also found that the strategies acquired during training were retained [44]. Further, in a complex rowing-type task, Sigrist et al. (2013) found that terminal feedback yielded greater retained improvements than concurrent feedback during multi-day training due to poor retention of benefits from concurrent training [45].

However, the findings from this study also differed from some results for other types of tasks. Yamamoto et al. (2019) found that concurrent visual feedback yielded retained improvements for low-skilled learners completing a single session of training on a load-control task, while terminal feedback did not result in improvements [46]. Chang et al. (2007) studied joint mobilization and found that both concurrent and terminal visual feedback resulted in retained improvements after a single session of training, and that there was no significant difference between the effects of the two feedback types [59]. Our findings therefore add to a growing body of literature comparing the effects of concurrent and terminal feedback.

In an analysis of the effects of concurrent and terminal visual feedback on ankle co-contraction using electromyography (EMG) data from the same dataset used for this study, ankle stiffness increased when concurrent feedback was used but not when terminal feedback was used [60]. In combination with the results of this study, these findings imply that concurrent feedback was used to decrease sway and increase sway velocity while stiffening the ankles. In contrast, terminal feedback yielded significant decreases in sway and sway velocity without changes in ankle stiffness. When concurrent feedback was removed, ankle stiffness significantly decreased while sway increased, and when terminal feedback was removed, neither ankle stiffness nor sway significantly changed. As stiffening the ankles is generally considered to be a maladaptive strategy, together these analyses suggest that terminal visual feedback may present a viable alternative to concurrent visual feedback during a single session of balance training as terminal feedback yields sway improvements and avoids maladaptive ankle stiffening. While further research is needed to fully examine the effects of such training, these findings suggest that terminal visual feedback should be further investigated as an alternative to concurrent visual feedback; the use of terminal feedback would also avoid the need for specialized equipment such as large, stationary displays. A balance training technology leveraging terminal visual feedback may require only a single, ubiquitous mobile device such as a smartphone, making such systems easily accessible to most. Prior studies have demonstrated that feedback-based balance training completed outside of clinical settings has the potential to yield balance improvements similar to those achieved during clinical training supervised by a physical therapist [61,62,63,64]. Consequently, the terminal visual feedback employed in this pilot study should be studied further as an accessible alternative to supervised clinical training.

Limitations of this study included: a small sample size, analysis of a single maximal model, performance of a single balance exercise, and a single session of balance training. Future work should evaluate the effects of training over multiple sessions, investigate retention over longer periods of time, and assess the effects of training on fall risk. Such a longitudinal study might also compare the effects of home-based training with concurrent or terminal visual feedback to in-clinic training with a physical therapist. Additionally, future work could explore different terminal feedback displays beyond the stabilogram presented in this study (e.g., a stabilogram with color coding for velocity or various summary statistics). Finally, additional balance training exercises and participant populations should be evaluated in order to assess the generalizability of these results to other balance tasks and populations.

## 5. Conclusions

This pilot study compared the immediate effects of concurrent and terminal visual feedback for older adults completing a common balance exercise. Both types of feedback yielded sway reductions. However, only training with terminal visual feedback yielded small, short-term, single-task sway reductions following the completion of the training protocol, potentially due to an adaptive mechanism, while training with concurrent feedback may have increased participants’ reliance on external information. These preliminary results suggest that terminal visual feedback may be a viable alternative to concurrent visual feedback for use in at-home balance training technologies. As balance training technologies employing terminal visual feedback may be achieved using only a single ubiquitous mobile device such as a smartphone without need for specialized equipment such as a large, stationary display, these preliminary findings offer promise for simple, affordable, and accessible balance training devices. Future research may further support that terminal visual feedback offers the potential for improved training retention in addition to practical advantages such as supporting a broader range of balance exercises.

## Figures and Tables

**Table 1:** Study participant demographics.

	Age	Sex	Height (m)	Mass (kg)
Group 1: Concurrent First (N = 10)	70.4 (±3.0)	7 Female	1.64 (±0.10)	72 (±16)
Group 2: Terminal First (N = 9)	70.0 (±4.0)	5 Female	1.66 (±0.06)	72 (±9)

**Table 2:** Terms included in the final models for each feature and the significance of those terms. ‘Yes *’ denotes inclusion and significance as determined by *p* < 0.05, ‘Yes’ denotes inclusion without significance, and ‘No’ denotes exclusion.

Natural Log of Feature	Exponent on Trial Number (a)	Intercept (1)	Order	Block	TrialNumber ^a^	Concurrent Feedback Removed	Terminal Feedback Removed
Phi RMS	0.4	Yes	No	Yes *	Yes *	Yes *	Yes
AP RMS	0.5	Yes *	No	Yes *	Yes *	Yes *	Yes
ML RMS	0.1	Yes *	No	No	Yes *	Yes *	Yes
MV	0.1	Yes	No	No	Yes *	Yes *	Yes
PL	0.1	Yes	No	No	Yes *	Yes *	Yes
EA	0.3	Yes *	No	Yes	Yes *	Yes *	Yes
**Natural Log of Feature**	**Block:** **TrialNumber ^a^**	**Block:** **Order**	**Concurrent Feedback** **Removed:** **Order**	**Terminal** **Feedback** **Removed:** **Order**	**Age**	**Sex**	**Participant**
Phi RMS	No	No	No	No	No	Yes	Yes
AP RMS	No	No	No	No	No	Yes *	Yes
ML RMS	Yes *	Yes *	Yes	No	No	No	Yes
MV	Yes *	No	No	Yes	No	No	Yes
PL	Yes *	No	No	Yes	Yes	No	Yes
EA	No	No	No	No	No	Yes	Yes

**Table 3:** Effects of trial number and removal of feedback on the log of each feature during the first training block. Features include Phi RMS (degrees), AP RMS (degrees), ML RMS (degrees), MV (degrees/s), PL (degrees), and EA (degrees^2^). * denotes significance (*p* < 0.05).

Natural Log of Feature	Exponent on Trial Number (a)	Effect of Training with Concurrent Feedback		Difference in the Effects of Training with Terminal vs. Concurrent Feedback	
		*p*-Value	Estimate [95% CI]	*p*-Value	Estimate [95% CI]
Phi RMS	0.4	<0.001 *	−0.030 [−0.046, −0.014]	0.975	−0.000 [−0.047, 0.046]
AP RMS	0.5	<0.001 *	−0.038 [−0.052, −0.023]	0.752	0.007 [−0.037, 0.052]
ML RMS	0.1	0.004 *	−0.095 [−0.159, −0.031]	<0.001 *	0.099 [0.049, 0.149]
MV	0.1	0.012 *	0.044 [0.010, 0.078]	<0.001 *	−0.116 [−0.142, −0.090]
PL	0.1	0.004	0.052 [0.016, 0.087]	<0.001 *	−0.127 [−0.154, −0.010]
EA	0.3	<0.001 *	−0.073 [−0.115, −0.030]	0.927	0.005 [−0.109, 0.120]
		**Effect of Removing Concurrent** **Feedback**		**Effect of Removing Terminal** **Feedback**	
		***p*-Value**	**Estimate [95% CI]**	***p*-Value**	**Estimate [95% CI]**
Phi RMS	0.4	<0.001 *	0.092 [0.038, 0.146]	0.711	0.011 [−0.045, 0.066]
AP RMS	0.5	<0.001 *	0.121 [0.051, 0.192]	0.526	0.024 [−0.049, 0.096]
ML RMS	0.1	0.003 *	0.106 [0.036, 0.175]	0.573	−0.016 [−0.072, 0.040]
MV	0.1	<0.001 *	−0.170 [−0.207, −0.132]	0.251	−0.027 [−0.074, 0.019]
PL	0.1	<0.001 *	−0.185 [−0.224, −0.146]	0.209	−0.031 [−0.080, 0.017]
EA	0.3	0.003	0.149 [0.050, 0.248]	0.944	0.004 [−0.098, 0.106]

**Table 4:** Difference between the predicted log feature value at baseline and at the end of the first block. * denotes significance (*p* < 0.05).

Natural Log of Feature	After Training with Concurrent Feedback		After Training with Terminal Feedback	
	*p*-Value	Estimate [95% CI]	*p*-Value	Estimate [95% CI]
Phi RMS	0.910	0.004, [−0.062, 0.070]	0.022 *	−0.07, [−0.145, −0.010]
AP RMS	0.557	0.024, [−0.107, 0.059]	0.005 *	0.122, [−0.208, −0.037]
ML RMS	0.732	−0.014, [−0.097, 0.068]	0.752	−0.011, [−0.082, 0.059]
MV	<0.001 *	0.111, [−0.159, −0.063]	<0.001 *	−0.122, [−0.179, −0.065]
PL	<0.001 *	−0.117, [−0.167, −0.068]	<0.001 *	−0.130, [−0.189, −0.071]
EA	0.815	−0.015, [−0.140, 0.110]	0.013 *	−0.160, [−0.288, −0.031]

## Data Availability

The de-identified datasets generated and analyzed are available upon reasonable request to Kathleen H. Sienko.
